# Effects of Individual Health Topic Familiarity on Activity Patterns During Health Information Searches

**DOI:** 10.2196/medinform.3803

**Published:** 2015-03-17

**Authors:** Ira Puspitasari, Koichi Moriyama, Ken–ichi Fukui, Masayuki Numao

**Affiliations:** ^1^The Institute of Scientific and Industrial ResearchOsaka UniversityIbarakiJapan

**Keywords:** health information search, health search activity pattern, health topic familiarity, sequence of search activities

## Abstract

**Background:**

Non-medical professionals (consumers) are increasingly using the Internet to support their health information needs. However, the cognitive effort required to perform health information searches is affected by the consumer’s familiarity with health topics. Consumers may have different levels of familiarity with individual health topics. This variation in familiarity may cause misunderstandings because the information presented by search engines may not be understood correctly by the consumers.

**Objective:**

As a first step toward the improvement of the health information search process, we aimed to examine the effects of health topic familiarity on health information search behaviors by identifying the common search activity patterns exhibited by groups of consumers with different levels of familiarity.

**Methods:**

Each participant completed a health terminology familiarity questionnaire and health information search tasks. The responses to the familiarity questionnaire were used to grade the familiarity of participants with predefined health topics. The search task data were transcribed into a sequence of search activities using a coding scheme. A computational model was constructed from the sequence data using a Markov chain model to identify the common search patterns in each familiarity group.

**Results:**

Forty participants were classified into L1 (not familiar), L2 (somewhat familiar), and L3 (familiar) groups based on their questionnaire responses. They had different levels of familiarity with four health topics. The video data obtained from all of the participants were transcribed into 4595 search activities (mean 28.7, SD 23.27 per session). The most frequent search activities and transitions in all the familiarity groups were related to evaluations of the relevancy of selected web pages in the retrieval results. However, the next most frequent transitions differed in each group and a chi-squared test confirmed this finding (P<.001). Next, according to the results of a perplexity evaluation, the health information search patterns were best represented as a 5-gram sequence pattern. The most common patterns in group L1 were frequent query modifications, with relatively low search efficiency, and accessing and evaluating selected results from a health website. Group L2 performed frequent query modifications, but with better search efficiency, and accessed and evaluated selected results from a health website. Finally, the members of group L3 successfully discovered relevant results from the first query submission, performed verification by accessing several health websites after they discovered relevant results, and directly accessed consumer health information websites.

**Conclusions:**

Familiarity with health topics affects health information search behaviors. Our analysis of state transitions in search activities detected unique behaviors and common search activity patterns in each familiarity group during health information searches.

##  Introduction

The emergence of the e-patient has encouraged non-medical professionals (consumers) to be more proactive regarding health care education and health decision making. More consumers are using the Internet to support health information needs [1-5]. A number of support systems have been developed to provide access to consumer-friendly health information. However, searching for understandable health information on the Internet is difficult for most consumers because they are not familiar with the standard health and medical terminology used in health care publications [6-9]. Thus, difficulties arise when formulating queries and when trying to understand documents. Researchers and health care providers are working on consumer-based initiatives to resolve the communication gap problem. In particular, Soergel et al [9] proposed an “interpretive layer” design to assist consumers when formulating effective queries, finding and interpreting relevant health information, and applying the information in an appropriate manner. This interpretive layer design concept has been implemented in several consumer health systems, such as Health Information Query Assistant (HIQuA) system [10], MedSearch [11], MedicoPort [12], and Interactive Online Health Information Systems [13].

To further reduce the communication gap between consumers and health care professionals/health materials, several researchers have studied the familiarity and recognition rate of health terminologies among consumers [6,7]. For example, Zeng et al developed the Consumer Health Vocabularies (CHV) initiative project, which links the vocabulary of consumers to the terminology used by health care professionals and in health care materials [6]. By building on the CHV project, several studies have proposed predictive models for measuring the average familiarity of various consumer health vocabularies based on term occurrence in text corpora [14], demographics factors [15], and contextual features [16,17]. In attempts to provide more consumer-friendly health materials, other researchers have developed automated tools for assessing the readability of health texts by substituting difficult terms with easier synonyms and simplifying long sentences [18] or by comparing the terms appeared in a document and terms known by the user [19]. Another study to improve the availability of consumer-friendly information is the consumer health educational project by European Patients’ Academy on Therapeutic Innovation (EUPATI) [20].

Previous studies in the information search area have demonstrated the impact of topic familiarity on search behaviors [21-25]. Seekers who have greater familiarity with the search topic use more varied and specific vocabulary [21], perform specific search strategies [21,22], and have better search efficacy [23]. One approach for examining search behaviors is to analyze the search activities performed by seekers [22,23]. Several studies have addressed the activities involved in search tactics [26] and search strategies [27,28]. To obtain a more comprehensive understanding, researchers have also studied the transitions among states during search activities [29-31] and analyzed the sequence of search activity transitions using state transition network [23] and Markov chains [30,32,33].

Most studies of health information search by consumers have focused on improving the health search experience of consumers by providing intelligent assistance and utilizing more consumer-friendly terminology. Several studies have also examined the perceived familiarity of health terminology among groups of consumers [14-17]. However, there is a lack of research on *individual* health topic familiarity and how this familiarity influences health information search behaviors in *specific* consumers. These research topics are important because every consumer has different health topic familiarities. For example, a consumer may be well informed about “skin allergy” but uninformed about “cardiovascular disease”, whereas another consumer may have the opposite health topic familiarities. The term “gastroesophageal reflux disease” may be well understood by some consumers, but completely unfamiliar to other consumers. This diversity may lead to misunderstandings because the information presented during health information searches may not suit the consumer’s level of familiarity.

Given the challenges of health search, a personalization approach based on the consumer’s familiarity is required to improve the search process. We consider that the familiarity has a larger impact on the search process (eg, the chosen search strategy/tactics, the performed search activity pattern) than on the search outcome (ie, the final information found). Searchers can find the correct information from many sources on the Internet that fits their needs. However, the process of finding the correct information is different among the searchers because it reflects their understanding about the health topic. It is expected that the unfamiliar searchers who had never heard of the search topic before would take a longer route and time to find the correct information and would face difficulty in the search process. These searchers need to build their understanding with the search topic first before they can locate relevant information. On the other hand, the familiar searchers would use advanced strategies and take a shorter route to find the correct information.

Thus, the purpose of this paper is to examine the effects of health topic familiarity on health information search behaviors by identifying the common search activity patterns exhibited by different groups of consumers with different levels of familiarity, which ranged from unfamiliar to familiar. The outcomes of this study will contribute to the improvement of the health information search process by providing suitable support for each searcher and by facilitating the development of a more advanced personalized health information search system.

## Methods

### Participants

In this study, the participants were observed in an experimental setting. A controllable environment and standardized health information search tasks are required to examine the effects of different parameters on the behaviors of participants. A convenience sample of 40 participants was recruited from several departments of a university in this study (Table 1). The participants were undergraduate students, exchange students, graduate students, and researchers from the Engineering, Material Physics, Applied Physics, Biotechnology, Information and Physical Sciences, and Computer Science departments. The criteria required for the recruitment of participants were non-medical professionals, the ability to read and write in English, and age ≥18 years. All participants had experience in health information searches on the Internet before the study was conducted.

**Table 1:** Demographic profiles of the participants.

Demographic profile	Categories	n	%
**Gender**			
	Male	24	60
	Female	16	40
**Age**			
	18–25 years	28	70
	26–35 years	12	30
	36–45 years	0	0
	> 45 years	0	0
**Native language**			
	English	15	38
	Non-English	25	62
**Education**			
	High school	0	0
	Bachelor’s degree	22	55
	Graduate degree	18	45
**Health information seeking experience**			
	Frequently on daily / weekly basis	8	20
	Occasionally on monthly basis	7	17
	Yearly or less than five times ever	5	12
	As the need arises	20	50
	Never	0	0

### Instruments

#### Overview

The instruments used for data collection comprised a health terminology familiarity questionnaire and a health information search task. The terminology questionnaire facilitated the rapid estimation of the familiarity of participants with predefined health topics, and the search task aimed to determine their search behaviors. Both instruments considered similar health topics, that is, skin allergy and its main treatments, cardiovascular disease, a common medical test (urinalysis), and cholesterol problems. The health topics selected for this study were based on the common health topics discussed on Yahoo Health [34] to ensure that the experiment reflected real-life health information searches. The answers for the entire health questions in this search task can be found in general consumer health informatics websites, health community/medical association websites, and medical journals listed in PubMed. We expected that the participants would be able to answer the questions easily. The participants can choose the correct answer from any sources according to their preference (familiarity).

#### Health Terminology Familiarity Questionnaire

**Figure 1:**
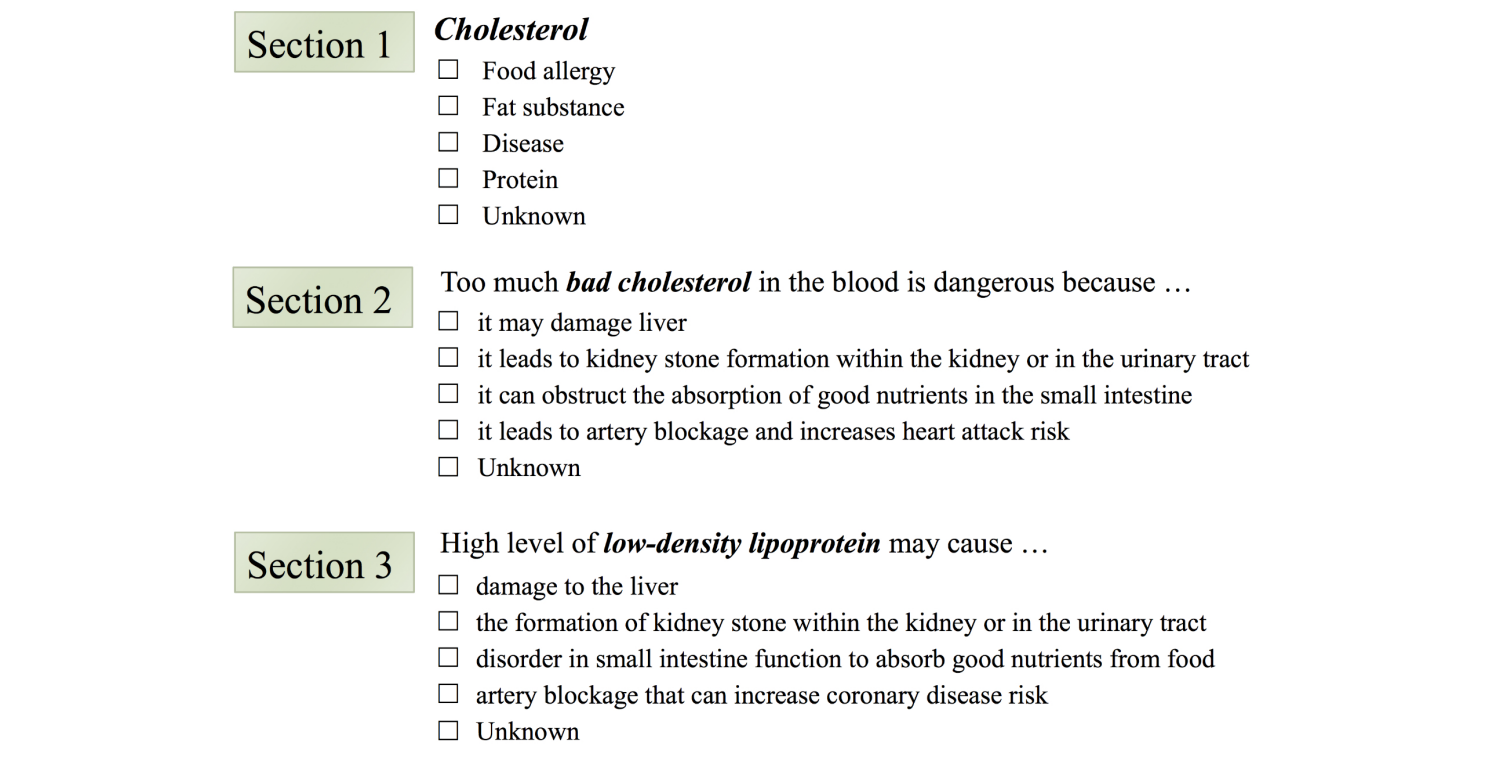
Examples of the questions included in the health terminology familiarity questionnaire.

The terminology questionnaire was modeled on the basis of the Familiarity of Sample Terms Questionnaire [14], the CHV Health Vocabulary Questionnaire [15], and the Test of Functional Health Literacy in Adults (TOFHLA) [35]. The questionnaire comprised three sections, each of which addressed the same four health topics. There were eight questions in each section. The questions with the same number in each section were equivalent (see Figure 1). The entire questionnaire is available in Multimedia Appendix 1. Section 1 estimated recognition at the surface level, while Sections 2 and 3 estimated the conceptual understandings of consumer-friendly terminology and the conceptual understandings of advanced health terminology, respectively. Each correct answer in the questionnaire was awarded 0.15 points for Section 1 and 0.175 points for Sections 2 and 3. The familiarity label was assigned to each health topic for each participant based on the total points awarded for the health topic (six questions). The labeling method employed in this study modified and extended previously described familiarity types [15], as follows: (1) Label L1 (unfamiliar) was assigned to a participant with total points ≤0.3 and label estimated that a participant had never heard of the terminology before or recognized it only at the surface level, (2) Label L2 (somewhat familiar) was assigned to a participant with total points >0.3 and ≤0.65, and estimated that a participant had some familiarity to associate the consumer-friendly health terminology with the basic phrase defining the terminology, and (3) Label L3 (familiar) was assigned to a participant with totals points >0.65 and estimated that a participant had good familiarity to associate the consumer-friendly terminology and its corresponding advanced terminology with the basic phrase defining the terminology.

#### Health Information Search Tasks

In this study, the health information search task comprised four separate tasks, each of which simulated one of the predefined health topics found in the questionnaire. A short scenario was added to each task to provide context (see Table 2).

**Table 2:** Health search tasks.

Task ID	Task description
Task 1	During the past six days, your skin has been very itchy and dry, particularly on your arm, wrist, and leg areas. You also noticed the appearance of rashes and redness on your itchy skin. You want to find out what might happen to your skin and how to treat it.
Task 2	In a first aid training course, your instructor emphasizes that lay people need to understand sudden cardiac arrest (SCA). SCA is often equated incorrectly with a heart attack, but SCA victims can survive if they receive treatment within 3-5 min after they collapse. You want to know (1) the difference between a heart attack and an SCA, and (2) how a lay person can help a victim when a suspected SCA incident happens in a public area.
Task 3	Every year your institution holds a mandatory general medical check-up. One of the medical tests is urinalysis. You usually receive the results about 3 weeks after the test. You want to know the purpose of each parameter (why each parameter is tested) in the sample below and the meaning of the results (normal or abnormal). Specific gravity: 1.030 (reference interval: 1.002-1.030) pH: 4.9 (reference interval: 4.6-7.5) Protein: Negative (reference interval: negative) Glucose: 100 mg/dL (reference interval: negative)
Task 4	Your doctor prescribed simvastatin and instructed you not to consume the medicine with grapefruit juice. You want to know the purpose of simvastatin and why it should not be consumed with grapefruit juice.

### Data Collection Procedure

The data were collected in a private laboratory. On arrival, the participant was welcomed and given a brief introduction to the purpose of this study, instructions on how to complete the questionnaire, and the procedure of the search tasks. The participants were also asked to review a consent form. Each participant performed the data collection process in the following order.

1. Demographic profile survey: The participant provided demographic information and details of their experiences with health information search on the Internet.
2. Health terminology familiarity questionnaire: The participant completed the questionnaire from Sections 1 to 3 in chronological order. If the participant had never heard of the term used in the question, the participant was requested not to guess the answer and instead they were asked to select the option “Unknown”.
3. Health information search task session: The participant was asked to complete the search tasks one by one. The participant was free to use any search engines or health information retrieval systems, to access any relevant websites, and to search at their own speed. Videos of all the search sessions were recorded using Camstudio screen and audio recording software [36].

After completing each task, the participant provided comments about the search topic and the search session.

### Data Analysis

#### Overview

The data collected from the participants comprised demographic data, responses to the familiarity questionnaire, and video recordings of the health information search sessions. Each participant produced four data instances, that is, one for each health topic. The demographic data were used to capture the general characteristics of the participants. The responses to the familiarity questionnaire were used to label the familiarity of participants with the predefined health topics. The participants were categorized into three familiarity groups (L1, L2, and L3). The search outcome (participant’s answer) from health information search task session was measured as relevant (correct) or not relevant to the question. Because this paper focused on the search process, we analyzed further only the search session from the Health Search Task that contained the finding of the relevant answer. Subsequently, the qualified video data were transcribed and analyzed.

#### Modeling Search Activities

This study used a search activity as the unit of analysis. A search activity comprised an action, which included an operational move and a conceptual strategy that the participants used to achieve their goal during the health information search process. A coding scheme was developed to transcribe the video data into a sequence of search activities. The overall coding scheme comprised 18 types of search activity, which were employed in the querying, evaluating, accessing, using, and discarding stages (see Table 3). Five types were modified from the study reported by Xie and Joo [30]: “Examining the retrieval result (E:ExamSR)”, “Evaluating the selected item (webpage) (E:EvalI)”, “Exploring link forward (A:XplorF)”, “Accessing link backward (A:AccB)”, and “Using the information (Use)”.

**Table 3:** Coding scheme for search activities.

Stage	Search activity code	Description
**Querying**		
	Q:AccSE	Access a general search engine/information retrieval system as the starting point during a health information search session.
	Q:AccHW	Access a consumer health informatics website as the starting point during a health information search session.
	Q:NewQ	Issue a new query, which is usually the first query in the search session.
	Q:ModQ	Reformulate the previous query to obtain more general/specific retrieval results.
**Accessing**		
	A:SelHI	Select and access a retrieved item from a health/medical website.
	A:SelGI	Select and access a retrieved item from a general/non-health-specific website.
	A:XplorF	In the retrieved item selected, access a link to another webpage that has not been visited before.
	A:AccB	Access a previously visited webpage using the browser’s back button, by following hyperlinks, or by tracking the history.
**Evaluating**		
	E:ExamSR	Examine the results retrieved to identify items (webpages) that contain potentially relevant health information.
	E:DisSR	Discard the results retrieved with or without examining their relevance.
	E:EvalI	Evaluate the selected item from the retrieved results or visit a webpage to determine its relevance.
	E:FindQ	Search for a specific keyword on a visited webpage.
**Using**		
	U:UseHI	Assess the visited health/medical webpage as a relevant source and use the information it contains to answer the questions in the search task.
	U:UseGI	Assess the visited general/non–health-specific webpage as a relevant source and use the information it contains to answer the questions in the search task.
**Discarding**		
	D:DisHI	Assess the visited health/medical webpage as an irrelevant source.
	D:DisGI	Assess the visited general/non-health-specific webpage as an irrelevant source.
	D:UnchkHI	Discard the selected health/medical webpage without visiting and evaluating its relevance.
	D:UnchkGI	Discard the selected general/non–health-specific webpage without visiting and evaluating its relevance.

To begin the health information search session, a participant accessed a general search engine (Q:AccSE) or visited a known consumer health website (Q:AccHW). Their familiarity with health topics may have influenced the starting points they selected. Next, the coding scheme included submitting a new query (Q:NewQ) and reformulating a query (Q:ModQ) because the query keywords and the type of query (new or modify) may have reflected the searcher’s information base, such as background knowledge and their familiarity with the search topic. During the evaluation stage, the participants exhibited different behaviors in terms of examining the search results (E:ExamSR) and evaluating an individual item (E:EvalI); thus both evaluation types were included in the coding scheme. When examining the search result, the searchers could not select a specific item/document from the results retrieved (E:DisSR). The evaluation stage also involved finding the query keyword (E:FindQ) because it may have indicated an advanced evaluation strategy or difficulty understanding the content. In the accessing stage, selecting an item from the results retrieved was included because it reflected the searcher’s ability to locate a potentially relevant source. The item selection was divided into two codes: selecting a result from a health/medical specific website (A:SelHI) and selecting a result from a general website (A:SelGI), considering that the familiarity with the search topic may influence the domain type selected. The next codes, that is, exploring forward (A:XplorF) and accessing backward (A:AccB), were treated as different codes because the direction of accessing has different meanings in the search process [30]. The next stages, that is, using and discarding, were included to study the participant’s behavior when assessing the webpages they visited, and to determine the efficiency and the success/failure rate of the overall search process.

After processing all qualified video data, each search session was encoded as a sequence of search activities. For example, a search session from a participant in a health search task exhibited nine search activities, as follows: the participant started the search session by accessing a general search engine, submitting the first query, and examining the results retrieved (Q:AccSE–Q:NewQ–E:ExamSR); the participant selected an item from a health website and an item from a non-health-specific website (A:SelHI–A:SelGI); the participant evaluated the first item selected and assessed whether it was a relevant source (E:EvalI–U:UseHI); and next, the participant evaluated the second item selected and assessed whether it was relevant (E:EvalI–D:DisGI).

Descriptive statistics were obtained to further examine the search activities performed by the participants in all of the familiarity groups.

#### Calculating the Transition Frequency Between Search Activity Types

To examine how the participants progressed during their search process, the next step involved calculating the transition frequencies and the probabilities between the states of all possible search activity types. Given a collection of mutually exclusive states (such as the search activity types in this study), the first-order transition probability in a Markov model gives the probability of moving from one state to another [32]. In this study, the transition probabilities were calculated on the basis of a first order Markov model.

**Figure 2:**
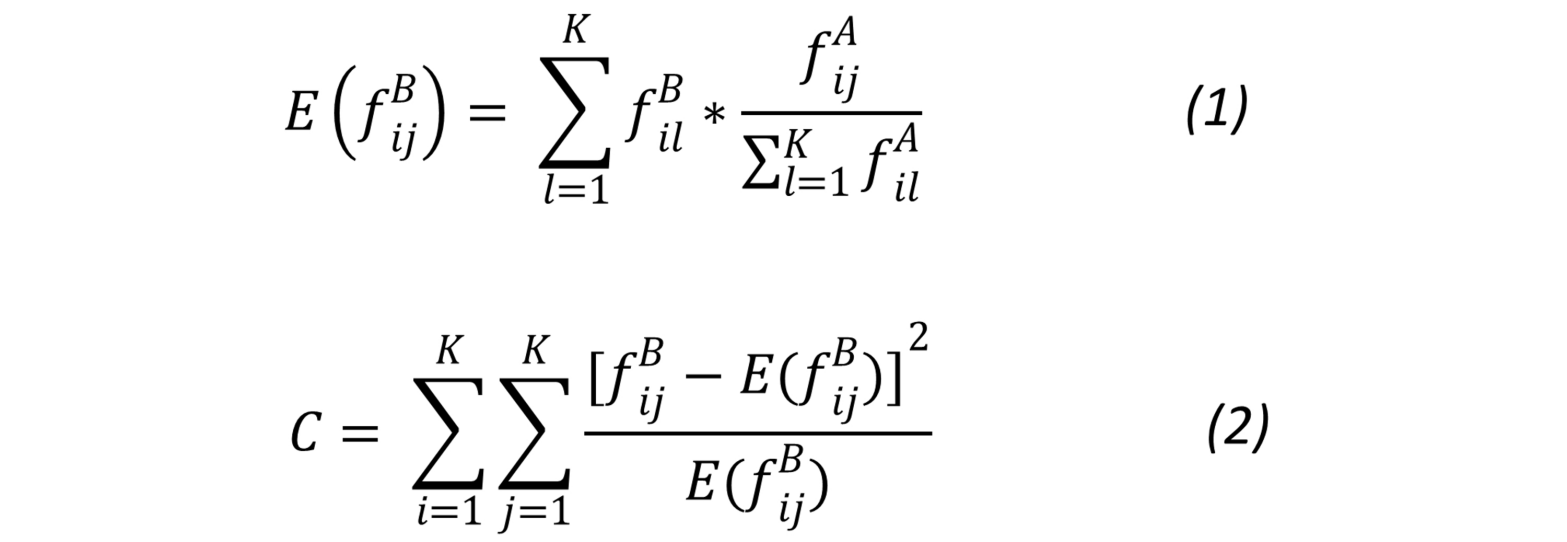
Equation (1) calculates the expected number of transitions from state i to state j in sample B. Equation (2) calculates the chi square score.

After calculating the transition frequency and probability for each familiarity group, the chi-square test was performed at a significance level of alpha=.01 to verify the differences in the search activity transitions between familiarity groups. The null hypothesis was that there was no difference in the first order state transition probability matrices between familiarity groups. The test followed the procedure reported by Chen and Cooper [32], as follows:

1. Let A and B be the two samples that need to be compared. A transition frequency matrix for sample A is defined as *f_ij^A (i,j=1, 2, …, K)*, where *f_ij^A* is the number of transitions from state *i* to state *j*, and *K* is the number of states in the state space.
2. If sample B is similar to sample A, then *f_ij^B* should be close to the expected number of transitions from state *i* to state *j* in B, as shown in Equation (1) of Figure 2.
3. In this case, the value *C* obtained from Equation (2) of Figure 2 will approximate a chi-square distribution with degrees of freedom: *K* ^*2*^ *− N*_*1*_ *− N*_*2*_, where *N*_*1*_ is the number of actual states in B and *N*_*2*_ is the number of impossible transitions in B. The null hypothesis that there is no difference between transition probability matrices A and B is accepted if C is less than the critical value of *C*_*a*_ *^(K* ^*2*^ *-N*_*1*_ *-N*_*2*_ *)* at a significance level of alpha=.01.

#### Identifying Search Activity Patterns

**Figure 3:**
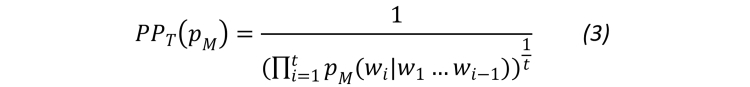
Perplexity equation.

To better understand and characterize the search behaviors of different familiarity groups, the next step in the data analysis process was to discover common search activity patterns using the following method:

1. Building an n-gram language model of the sequence of search activities performed by participants based on the dataset. An n-gram model is a probabilistic language model, which is used to predict the next word from a sequence of word [37]. When estimating an n-gram model, it is normally assumed that the sequence histories of words depend only on the local prior context (Markov model assumption) because of the large number of parameters involved [33]. To build an n-gram language model, we utilized the SRI Language Modeling toolkit [38] and four datasets (L1, L2, L3, and the data for all participants) with the Witten-Bell discounting strategy [39]. Each dataset was divided into 80% training data and 20% test data. The n-gram language models were built using the training data with various sequences: 2-grams to 7-grams.
2. Evaluating the perplexity of the computed language models to specify the number of search activities in a sequence that best represented the search activity pattern. The perplexity of a language model represents the geometric average branching factor of the language according to the model and is used widely to measure the quality of a model (lower perplexity tend to have lower word-error rates) [40]. The perplexity *PP*(*p*_*M*_) of a language model *p*_*M*_ (next word *w*|history *h*) on a test set *T*={*w*_*1*_ *, …, w*_*t*_} is computed using the equation in Figure 3. This metric was used because the computed language models contained similar vocabularies (ie, the search activity types). The number of search activities in a sequence was represented by the n-gram sequence with the lowest perplexity.
3. Applying the selected n-gram model to the sequence of search activities in the datasets to identify common search activity patterns.

## Results

### Health Topic Familiarity of the Participants

Table 4 shows the result of familiarity labeling for each health topic based on the responses to the familiarity questionnaire. According to this result, each participant in this study could have different familiarity labels for different health topics. For example, a participant could be highly familiar with topics 2 and 4, but unfamiliar with topics 1 and 3.

**Table 4:** Results of familiarity labeling for each health topic.

No.	Health topic	L1	L2	L3	Participants, n
1	Skin allergy and main medications	14	9	17	40
2	Cardiovascular disease	12	19	9	40
3	Common medical test (urinalysis)	17	11	12	40
4	Cholesterol problems	18	12	10	40
	Total	61	51	48	160

### Frequency of Search Activities

**Figure 4:**
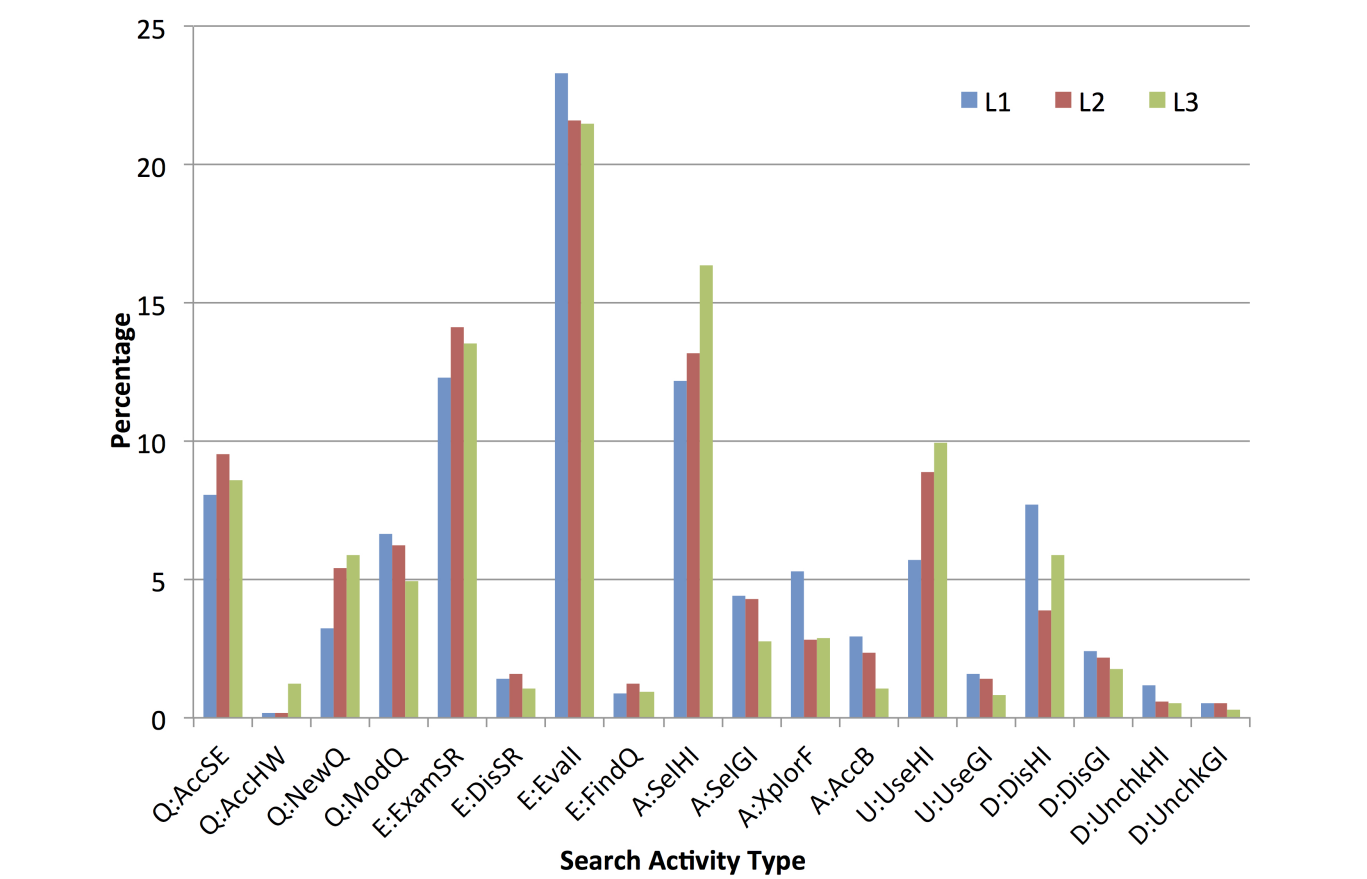
Percentage of the search activity types in all familiarity groups.

All of the search sessions performed by the 40 participants contained finding the correct answer to the questions in a Health Search Task. Thus, all of the video data were transcribed and produced 4595 search activities (Figure 4 and Multimedia Appendix 2). The number of search activities in a health information search session varied from 6 to 221. On average, a participant performed 28.7 search activities during one health information search session (SD 23.27).

The most frequent search activity in all the familiarity groups was evaluating a selected item from the results retrieved (E:EvalI). This search activity accounted for 562 out of 2424 (23.31%) activities in group L1, 260 out of 1204 (21.59%) in group L2, and 208 out of 967 (21.51%) in group L3. The second, third, and fourth most frequent search activities in groups L1 and L2 were examining the results retrieved (E:ExamSR), selecting a health-related item from the results retrieved (A:SelHI), and accessing a general search engine (Q:AccSE), which together comprised 32.55% (789/2424) of the activities by group L1 and 36.88% (444/1204) by group L2. In contrast to these groups, A:SelHI, E:ExamSR, and U:UseHI were the second, third, and fourth most frequent search activities among participants in group L3, which together represented 39.81% (385/967) of the total. The fifth most frequent search activities were discarding the selected health-related website (D:DisHI), U:UseHI, and Q:AccSE for groups L1, L2, and L3, respectively.

All of the groups exhibited the same pattern when accessing the results retrieved. Participants were more likely to access health/medical websites than general domain websites. Group L3 accessed health websites more frequently than others, 85.42% (158/185) compared with 73.4% (159/211) and 75.36% (295/402). In contrast, group L1 accessed more general domain websites (26.60%, 107/402) than group L2 (24.64%, 52/211) and group L3 (14.58%, 27/185). In terms of locating the relevant health information, the participants in all groups tended to engage in a considerable number of search activities before reaching U:UseHI or U:UseGI. The combinations of U:UseHI and U:UseGI in groups L1, L2, and L3 were 7.30% (177/2424), 10.30% (124/1024), and 10.75% (104/967), respectively.

### Transition Between Search Activity Types

Table 5 provides most frequent transitions between search activities. The calculations yielded a total of 4435 transitions, that is, 2363 transitions, 1153 transitions, and 919 transitions in groups L1, L2, and L3 respectively. The average numbers of transition between two search activities were 19.86 (SD 24.70) in group L1, 14.06 (SD 13.26) in group L2, and 11.78 (SD 11.38) in group L3. The most frequent transitions in all groups were related to accessing a health website from the results retrieved and evaluating its relevancy. The corresponding transitions were from E:ExamSR to A:SelHI (L1=7.96%, L2=9.80%, L3=11.1%) and from A:SelHI to E:EvalI (L1=7.66%, L2=8.76%, L3=11.0%).

The third most frequent transition in the unfamiliar group (L1) was different from that in the other more familiar groups (L2 and L3). The transition in group L1 from E:EvalI to D:DisHI showed that the participants assessed the selected item as irrelevant. In contrast, the third most frequent transition in groups L2 and L3 was from E:EvalI to U:UseHI. This finding indicates that the participants in L2 and L3 were probably more successful than those in L1 at identifying potentially relevant items from the results retrieved.

During the querying stage, group L3 had different search activities compared with the other less familiar groups (L1 and L2). The most frequent transition related to the querying stage was from Q:NewQ to E:ExamSR in group L3 and from Q:ModQ to E:ExamSR in groups L1 and L2. This shows that the L3 participants probably relied on their first query to discover relevant results. Group L3 also performed fewer query modifications than the other groups.

**Table 5:** Top 10 frequent first order transitions for each familiarity group.

No.	L1			L2			L3		
	Transition	Frequency		Transition	Frequency		Transition	Frequency	
		n	%		n	%		n	%
1	E:ExamSR–A:SelHI	188	7.96	E:ExamSR–A:SelHI	113	9.80	E:ExamSR–A:SelHI	102	11.1
2	A:SelHI–E:EvalI	181	7.66	A:SelHI–E:EvalI	101	8.76	A:SelHI–E:EvalI	101	11.0
3	E:EvalI– D:DisHI	160	6.77	E:EvalI–U:UseHI	94	8.15	E:EvalI–U:UseHI	81	8.8
4	Q:ModQ–E:ExamSR	158	6.69	Q:ModQ–E:ExamSR	75	6.50	Q:NewQ–E:ExamSR	56	6.1
5	Q:AccSE–Q:ModQ	121	5.12	Q:NewQ–E:ExamSR	64	5.55	E:EvalI–D:DisHI	51	5.6
6	E:EvalI–U:UseHI	120	5.08	Q:AccSE–Q:NewQ	63	5.46	Q:AccSE–Q:NewQ	48	5.2
7	A:XplorF–E:EvalI	91	3.85	Q:AccSE–Q:ModQ	52	4.51	A:SelHI–A:SelHI	44	4.8
8	E:EvalI–A:XplorF	88	3.72	A:SelHI–A:SelHI	40	3.47	Q:ModQ–E:ExamSR	44	4.8
9	Q:AccSE–Q:NewQ	75	3.17	A:SelGI–E:EvalI	39	3.38	Q:AccSE–Q:ModQ	35	3.8
10	Q:NewQ–E:ExamSR	75	3.17	E:EvalI–D:DisHI	36	3.12	A:XplorF–E:EvalI	24	2.6
Total		1257	53.20		677	58.72		586	63.8

### Testing the Differences in Search Activities Between Familiarity Groups

Table 6 shows the result of the chi-square test described above. According to the results, the null hypothesis was rejected in all cases; hence, the three familiarity groups exhibited distinct search activity patterns.

**Table 6:** Results obtained after testing the differences between the familiarity groups (*P*<.001).

Familiarity group	L2	L3
L1	K^2^=324^a^	K^2^=324
	N_1_=18^b^	N_1_=18
	N_2_=242^c^	N_2_=246
	df=64 (χ^2^=104.716)^d^	df=60 (χ^2^=99.607)
	C=5084.883^e^	C=6021.407
L2	—	K^2^=324
		N_1_=18
		N_2_=246
		df=60 (χ^2^=99.607)
		C=2809.463

^a^K is the number of states in the state spaces.

^b^N_1_ is the number of actual states.

^c^N_2_ is the number of impossible transitions.

^d^df is obtained from K^2^-N_1_-N_2._

^e^C is the chi-square score obtained from Equation (2) of Figure 2.

### Most Frequent Patterns in Search Activity Sequences for Each Familiarity Group

**Figure 5:**
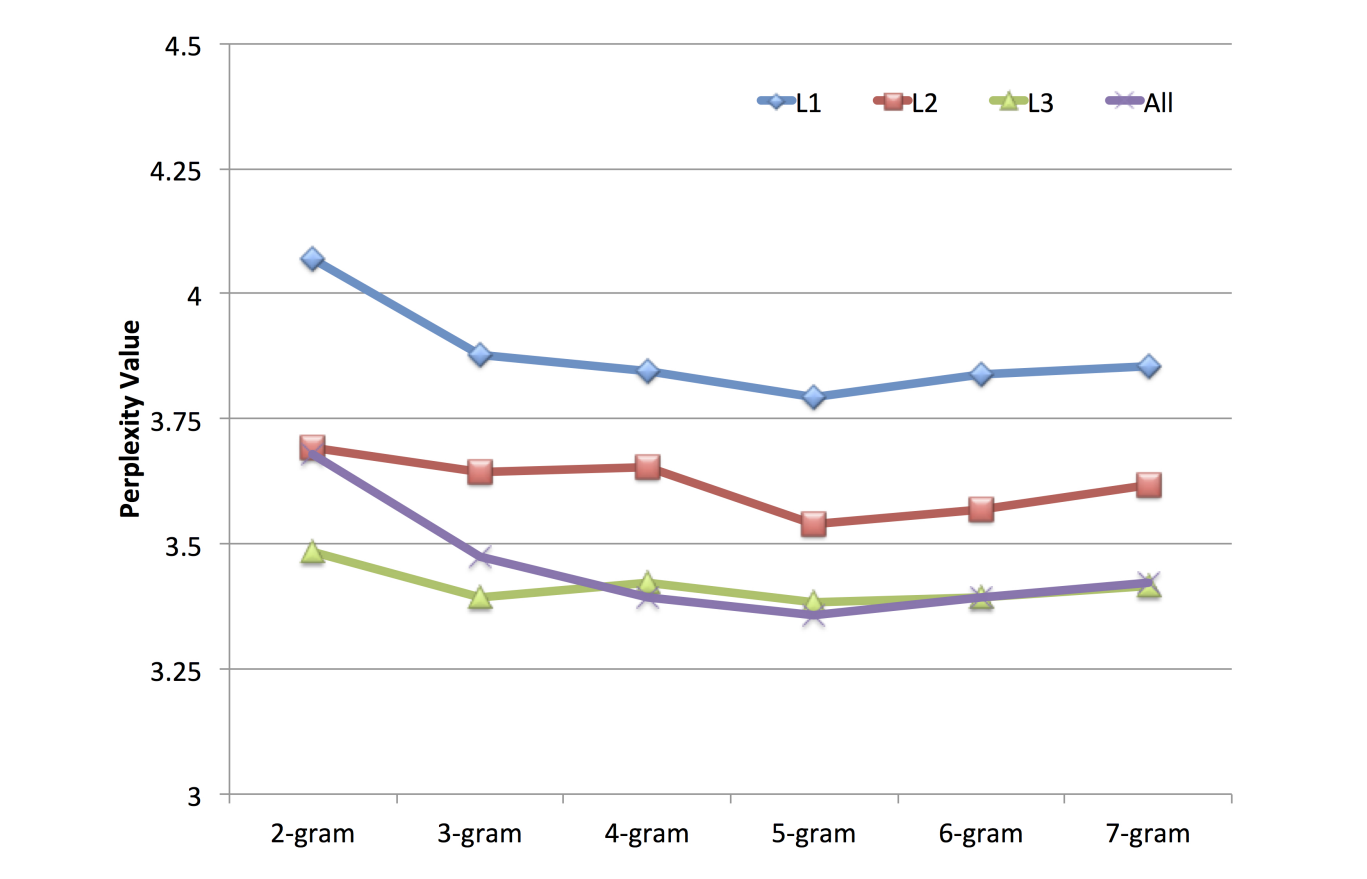
Perplexity values for L1, L2, L3, and all the test data using different n-gram models.

According to the perplexity evaluations of all the language models for all the datasets (Figure 5), 5-gram language models had the lowest perplexity values for the four test datasets. Thus, we used *5-*gram sequences to identify common search activity patterns in each familiarity group. The numbers of observed 5-gram sequences in groups L1, L2, and L3 were 940, 444, and 359, respectively. There were large numbers of 5-gram sequences in each group, so only the 20 most frequent sequences were examined (for details, see Multimedia Appendix 3). Above this level, the frequencies of the sequences were too low to represent the search activity patterns in a familiarity group.

**Figure 6:**
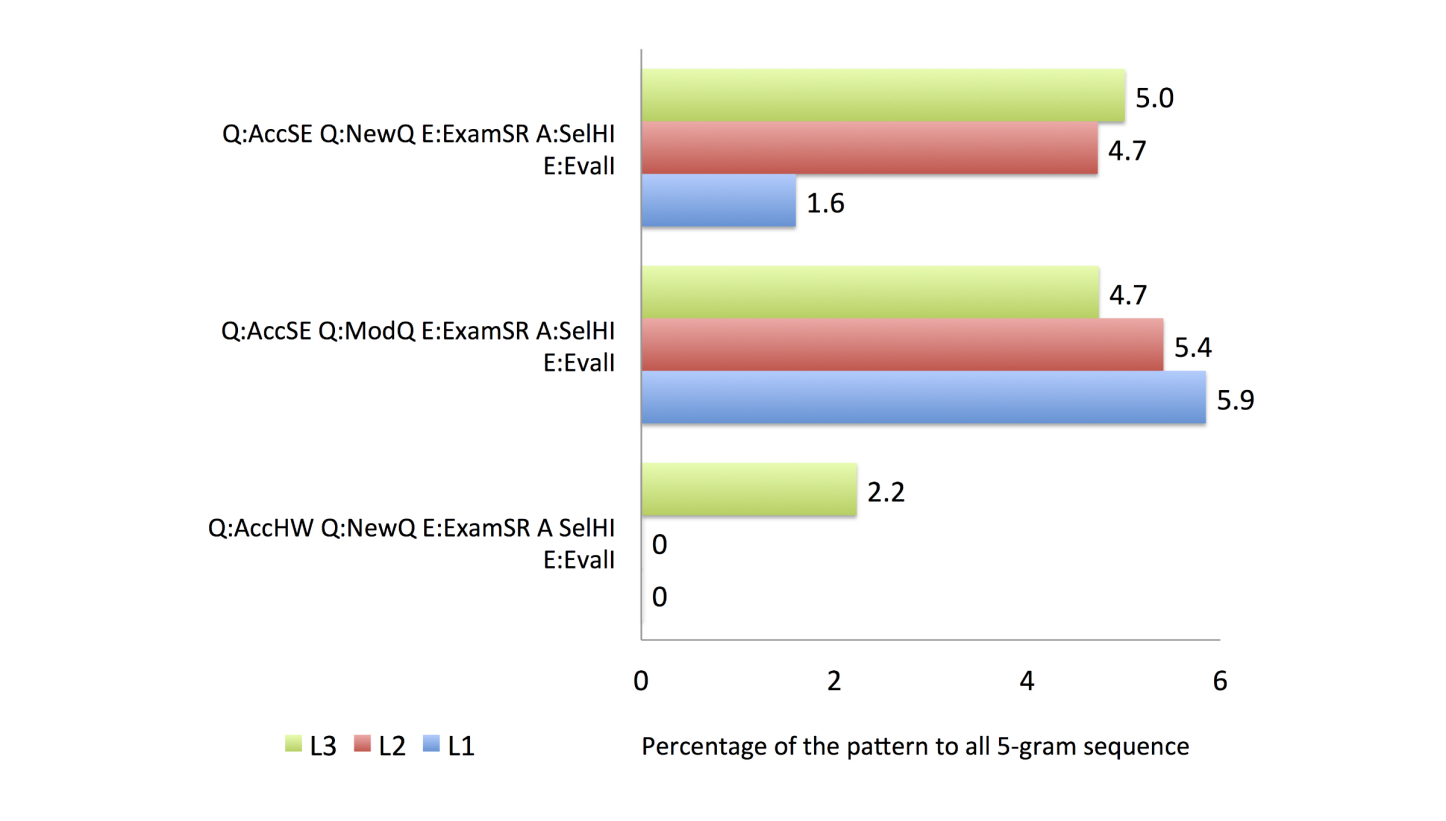
Comparison of frequent activity patterns in Category 1.

**Figure 7:**
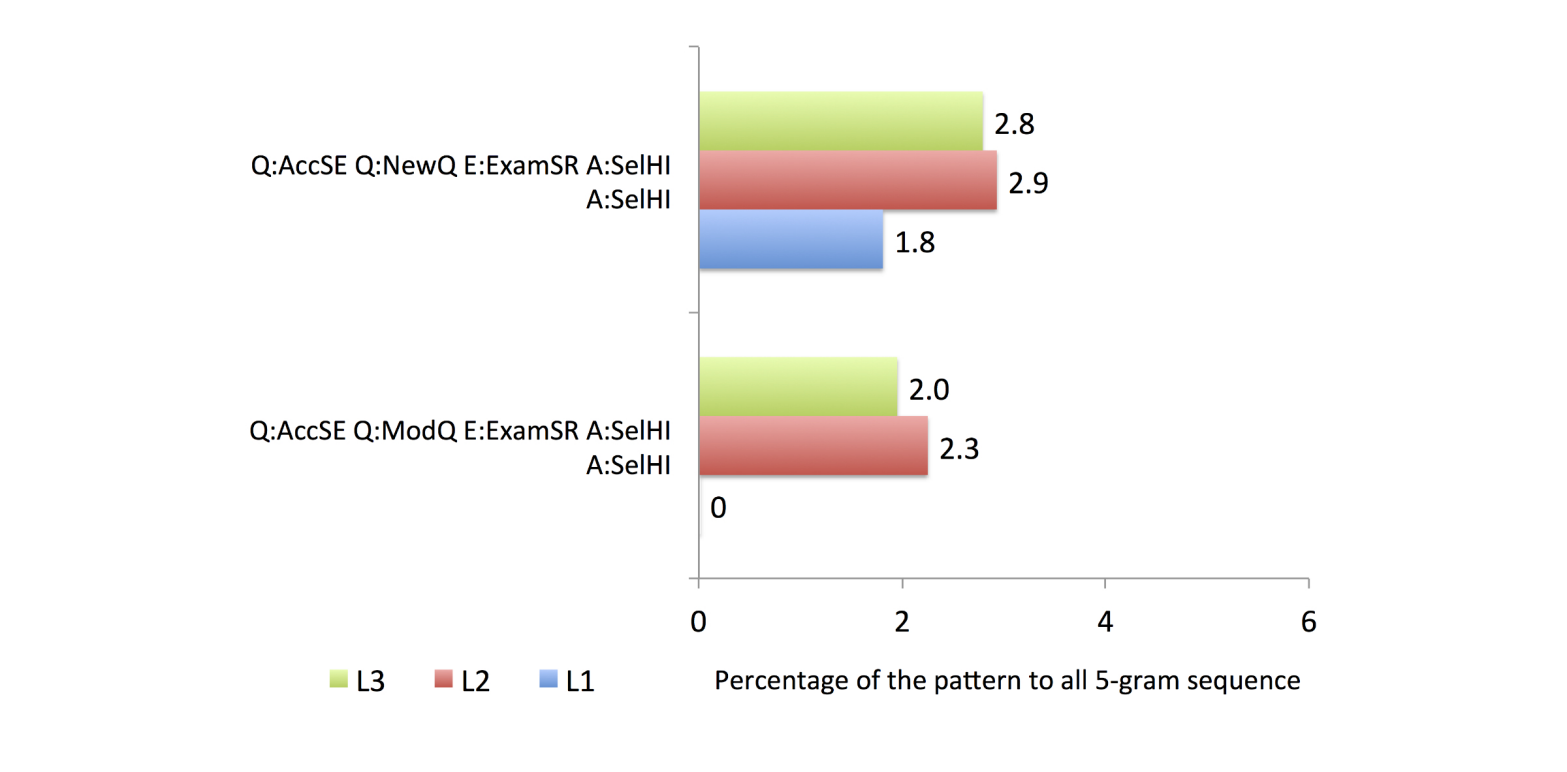
Comparison of frequent activity patterns in Category 2.

**Figure 8:**
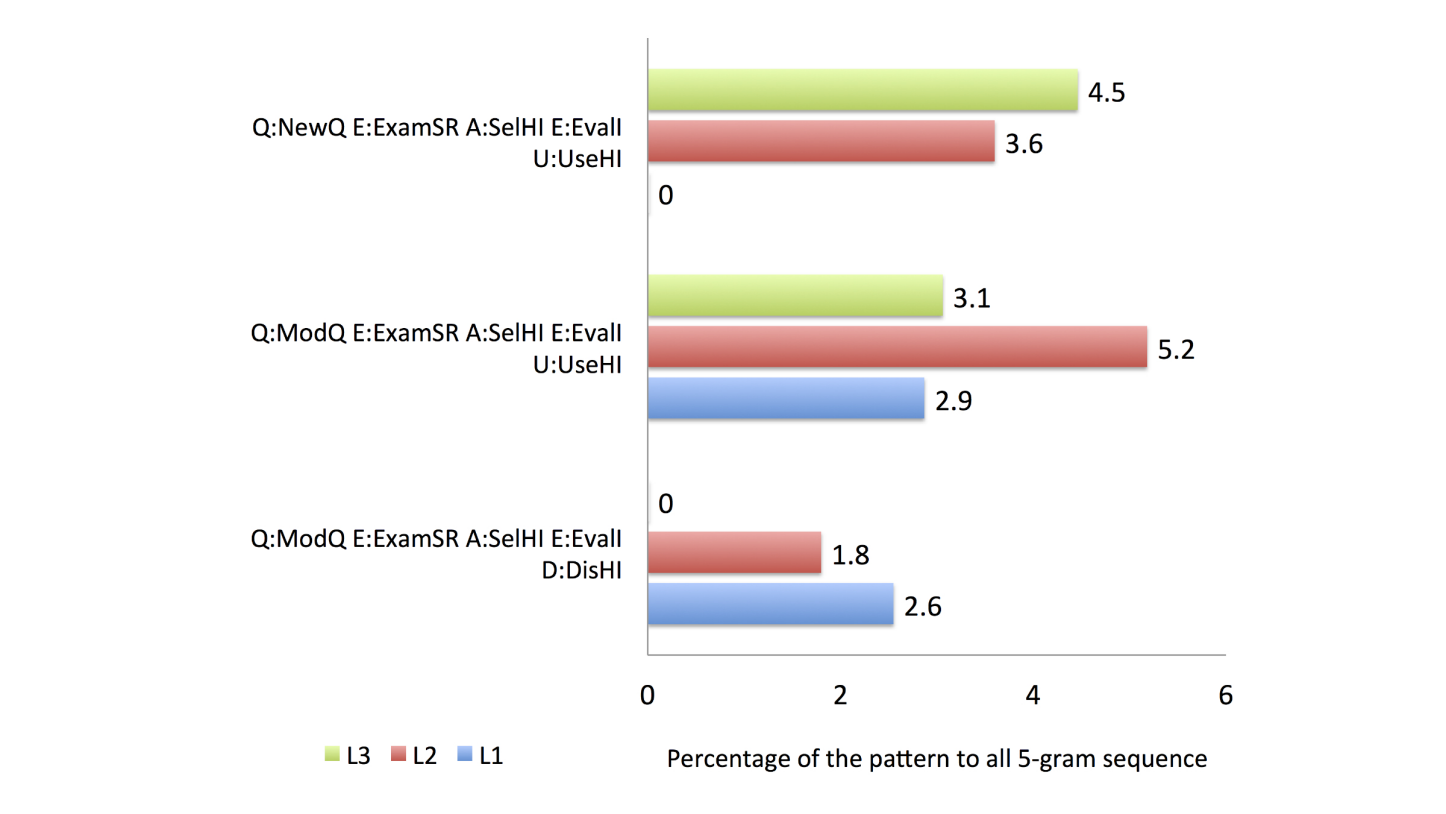
Comparison of frequent activity patterns in Category 3.

**Figure 9:**
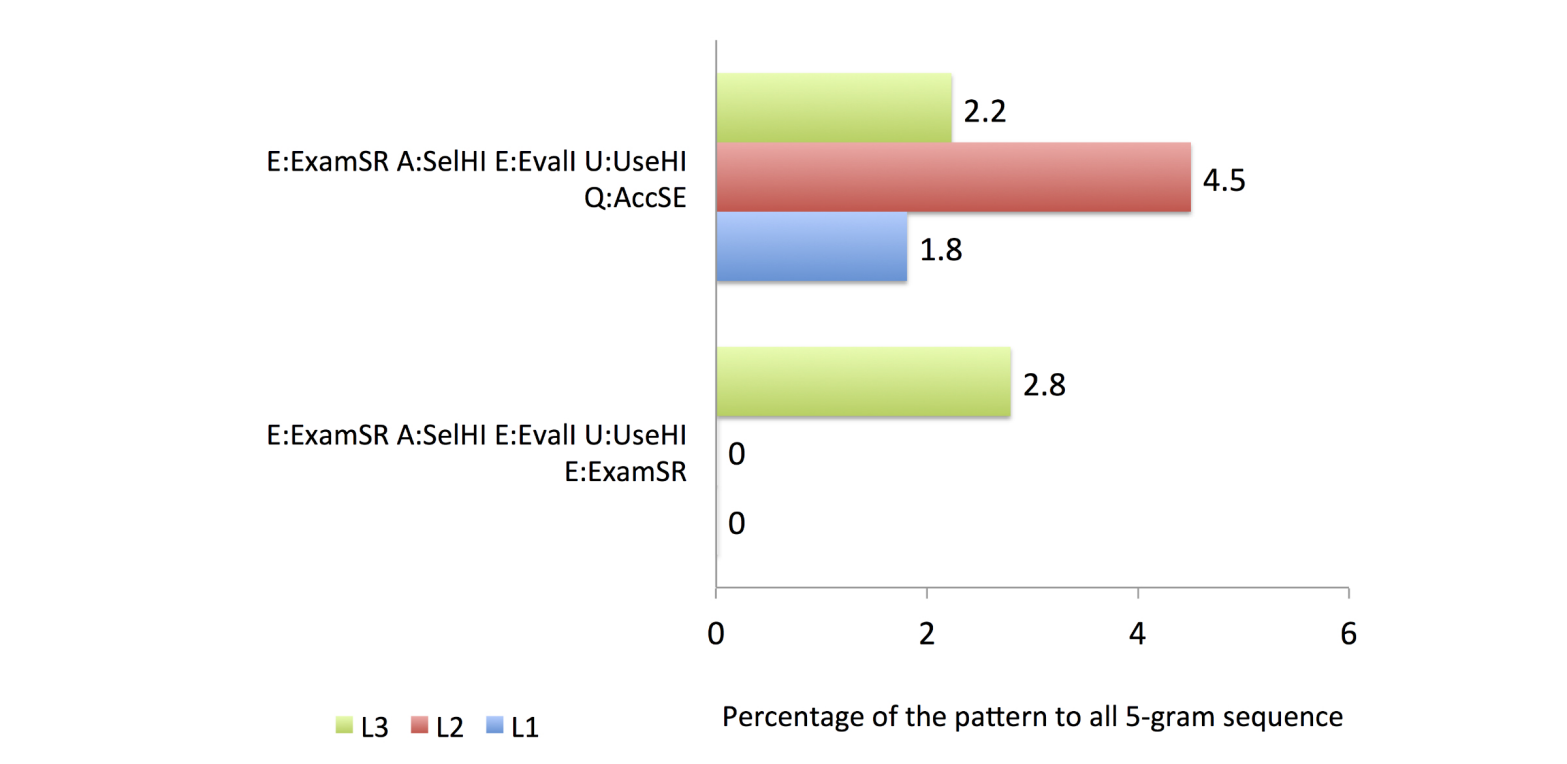
Comparison of frequent activity patterns in Category 4.

To compare the search behavior between familiarity groups, we used four activity categories from the health information search process based on the top 20 most frequent patterns, as follows: (1) the first category comprised accessing a search engine (general search engine or consumer health website), issuing a new or modified query, and accessing and evaluating an item from a health website (see Figure 6), (2) the second comprised accessing a search engine, issuing a query, and accessing multiple items from health websites (see Figure 7), (3) the third category was related to the assessment of the relevancy of the item selected from a health website (see Figure 8), and (4) the fourth category involved continuing the search process after finding a relevant item (see Figure 9).

Group L1 comprised participants who were not familiar with the health topic search task. The most frequent pattern in group L1 was submitting a modified query to a general search engine, followed by accessing a health-related website from the search results, and immediately evaluating the relevancy of the selected result (Q:AccSE–Q:ModQ–E:ExamSR–A:SelHI–E:EvalI), which accounted for 5.85% of all the 5-gram patterns. In locating the potentially relevant search results, this group accessed more non-relevant results than relevant results.

As shown in Figure 8, the proportion of D:DisHI assessments was larger than that of U:UseHI assessments. In total, 10/20 of the most frequent patterns contained D:DisHI (see Multimedia Appendix 3), which accounted for 23.3% of all the 5-gram patterns in group L1. In contrast, only 5/20 of the most frequent patterns included U:UseHI assessments, which comprised 11.5% of all the 5-gram patterns.

In group L2, all of the queries in the top 20 most common patterns were submitted to a general search engine. The proportion that issued a modified query was higher than that issuing a new query. The identification of the potentially relevant search results showed that participants in this group were likely to be more successful than those in group L1, as demonstrated by the higher proportion of U:UseHI assessments than D:DisHI assessments. The participants in group 2 created a new search after finding a relevant information source.

The final group, L3, had the most knowledgeable searchers. The proportion that issued a new query was higher than that issuing a modified query. Unlike the other groups, the participants in group L3 also accessed consumer health websites to search for health information. Two strategies were performed by group L3 when accessing the search results: accessing a single item from a health website and evaluating it immediately, or accessing multiple items from health websites and evaluating the items one by one. When identifying potentially relevant search results, group L3 found more relevant items in the results retrieved from the first query compared with the results retrieved using the modified query. The participants also continued their search process by creating a new search and reexamining the previous results retrieved.

## Discussion

### Summary

This study considered the concept of individual health topic familiarity, where we examined its effects on health information search behaviors. In previous studies, the effects of familiarity during health information searches were investigated mainly in the context of the health terminology recognition rate by *all* consumers. In this study, we shifted the concept of familiarity from the health terminology perspective to the consumer perspective. To the best of our knowledge, this study is among the first to examine the effects of health topic familiarity on the activity pattern during health information searches. Results and findings from this study show that familiarity with health topics affects search activity patterns exhibited during health information searches.

This discussion section is organized around two themes: (1) effects of the familiarity level of consumers on their health information search behaviors, and (2) the implications for health search systems of better support for individual familiarity with health topics.

### Effects of Level of Familiarity on Health Information Search Behaviors of Consumers

We characterized health information search behaviors as a sequence of search activities in this study. The frequencies of the search activities showed that the participants devoted substantial efforts during the evaluating stage, where they examined the results retrieved and evaluated the relevancy of the item selected. The participants also performed frequent search activities during the accessing and querying stages. Although the use of selected information (U:UseHI and U:UseGI) is the main goal of information search, the total proportions of these search activities were smaller than the search activities performed in the evaluating, accessing, or querying stages. These findings indicate that health information search remains difficult for most consumers.

In this study, the participants with different levels of familiarity performed a unique search behavior (see the summary in Table 7). The first effect of health topic familiarity was observed in the querying stage. The participants in the lower familiarity groups submitted more queries than the participants in the higher familiarity group. The average numbers of query submissions during a health search session were 7.2, 5.0, and 4.2 in groups L1, L2, and L3, respectively. The series of query submissions reflected the searcher’s progress in understanding the searched topic. The participants with less familiarity submitted more queries because they needed to increase their understanding of the search topic before they could locate relevant information. A number of participants in group L1 started the search process by searching for definitions of the health terms that appeared in the searching task. Examples of this type of query are “what is rash”, “urinalysis definition”, “what is SCA”, “special gravity in urine?”, and “what is simvastatin”. This finding is different from other studies in general Web-based search processes [41,42]. Liu et al in their study reported that no differences in the number of queries issued were found between users with different levels of topic knowledge [41], while Zhang et al stated in their study that the high-level domain knowledge group issued more queries than the low-level group [42]. In term of the average query length, there was no distinguishable pattern between less familiar and more familiar groups. This finding is also different from previous studies in [21,43] that suggested expert users issued longer and more complex queries than novice users.

Another interesting finding is how the familiarity affected the selection of the relevant source (webpages). Less familiar participants were likely to choose easier content, while more familiar participants tended to use more difficult content. We measured the difficulty of the source by its readability score using the Simple Measure of Gobbledygook (SMOG) formula [44]. We selected this formula because SMOG was the preferred measure of readability when evaluating consumer-oriented health care material [45].

The next effect was detected when locating relevant health information, which was estimated on the basis of the search efficiency. The search efficiency compared the proportion that used the information (U:UseHI and U:UseGI) against the number of items accessed (A:SelHI, A:SelGI, A:XplorF, and A:AccB). Group L3 achieved the best performance with a search efficiency of 46.6%, compared with 45.5% and 29.4% for groups L2 and L1, respectively. This result agreed with the frequencies of search activities in each familiarity group. Group L1 accessed more irrelevant items than relevant ones, whereas groups L2 and L3 did the opposite. This finding is in contrast to a previous study that reported that the search effectiveness remained the same for all participants in high and low levels of domain knowledge [42].

The patterns exhibited in each group also illustrated the effect of the level of health topic familiarity on search behaviors. The frequent patterns in group L1 showed that these participants were likely to experience difficulties during their health information search sessions, as demonstrated in the much higher percentage of issuing modified queries than issuing new queries and in identifying the potentially relevant search results. The participants found relevant information more often using the results retrieved with the modified query than the first query. The common strategies employed when the participants encountered search problems were querying followed by single accessing and evaluating (… D:DisHI–Q:AccSE–Q:ModQ …), or iterative accessing and evaluating (… D:DisHI–E:ExamSR–A:SelHI …).

In group L2, the most frequent pattern was issuing a modified query, accessing a health website, and evaluating the selected item immediately. Group L2 also discovered relevant items more often using the results retrieved with the modified query rather than the first query, but they exhibited greater search efficiency compared with group L1. When examining the results retrieved, group L2 performed single accessing and the evaluation of selected items, or multiple accessing followed by evaluating the selected items one by one. Another frequent pattern in group L2 was the transition from U:UseHI to Q:AccSE. This pattern indicates that the participants attempted to continue health information searches after they found relevant health information. The aim of these further searches was either to verify the accuracy of the health information they discovered, or to search for another related health topic during the search task.

The most common patterns in group L3 were related to query submission and single selection, and the evaluation of a health webpage. The participants in group L3 employed more varied keywords in their queries than the other groups. A frequent pattern in this group was accessing a known consumer health information website directly to start a health search session and search for health information (known item strategy). Several participants also referred to PubMed articles to answer the questions in the search tasks, for example, in Task 4 (the interaction between simvastatin and grapefruit juice). Another highly frequent pattern in group L3 was Q:AccSE–Q:NewQ–E:ExamSR–E:EvalI–U:UseHI, which represents a successful search when locating the relevant health information at the first attempt (first query submission and first item selection). A number of participants in group L3 continued the search process after they discovered relevant health information by issuing a modified query, or by reexamining the previous results retrieved.

**Table 7:** Summary of the findings.

Familiarity group	Characteristic frequent patterns
L1	More likely to reformulate the query: the proportion of frequent patterns that contained a modified query (Q:ModQ) was higher than that containing the first query (Q:NewQ).
	More likely to encounter difficulty during the search process, eg, they frequently accessed irrelevant websites and had a low search efficiency.
	Discovery of relevant webpages (information source) more frequently in the results were retrieved with the modified query than the first query.
L2	More likely to reformulate the query: the proportion of frequent patterns that contained a modified query (Q:ModQ) was higher than that containing the first query (Q:NewQ).
	Discovery of relevant webpages (information source) more frequently in the results were retrieved with the modified query than the first query.
	Achievement of better search efficiency than group L1.
	Continuation of the search process after discovering relevant webpages by issuing another query.
L3	Access of consumer health information websites directly to start the search session.
	Discovery of relevant webpages (information source) more frequently in the results were retrieved with the first query than the modified query.
	Continuation of the search process by issuing another query or by reexamining the results retrieved.

### Implications for Health Information Search Systems

The main finding of this study is the identification of unique search patterns between different familiarity groups (unfamiliar, somewhat familiar, and familiar). Health information search systems can use this knowledge to identify the term familiarity by analyzing consumer’s search behaviors. For example, multiple query reformulations pattern without any activities on the retrieved results may indicate unfamiliarity with the search topic. Addressing individual familiarity in health information search systems is necessary to provide better support for the consumers and to improve the overall search process.

To support unfamiliar consumers, these systems should implement assistive features during the construction of health queries and select understandable health information. These systems could help consumers build queries using predefined diagnosis questionnaires and/or human anatomy diagrams. To support unfamiliar searchers with the identification of potentially relevant results, these systems should automatically extract a consumer-friendly definition of the submitted health query, adjust the rankings of the items retrieved, and suggest a related term using CHV. For more familiar searchers, these systems could be of assistance by locating additional relevant results. Based on the patterns exhibited in this study, groups L2 and L3 were likely to continue the search process after they discovered relevant information. Systems could assist this process by clustering similar items into topic clusters in the page showing the results retrieved, by adjusting the ranking of retrieval items, and by providing a summary of health topic keywords.

### Limitations and Future Studies

Most of the results obtained in this study correspond to our goals, but a more comprehensive user study is required for further validation. First, the participants involved in this study shared several common demographic characteristics, that is, higher education and a high level of experience in using the Internet. Therefore, the generalizability of the results is limited. A future user study should investigate further the background of the participants. Second, the time spent examining the results retrieved and evaluating the selected webpages were not considered in the search activities model. The time variable may characterize the search behaviors of different familiarity groups, and it needs to be considered in future studies.

The findings of this study may facilitate the development of a more advanced personalized health information search system based on the individual’s health topic familiarity. This type of system could identify the consumer’s familiarity with health topics by analyzing their usage behavior to provide suitable support. Because health information search remains challenging for most consumers, this approach would be a major improvement in health information search systems.

### Conclusion

This study addressed the concept of individual familiarity with health topics and investigated its effects on health information search behaviors. The results of this study support two main conclusions. First, the analysis of state transitions in search activities can detect the unique behaviors of consumers in each familiarity group during health information searches. Second, we identified common health search patterns in unfamiliar and familiar groups. These patterns characterized the familiarity groups during all stages in health information searches.

## Multimedia Appendix 1

Health terminology familiarity survey.

## Multimedia Appendix 2

Frequency and proportion of search activity type.

## Multimedia Appendix 3

Most frequent 5-gram sequence patterns.

## Abbreviations

CHV: Consumer Health Vocabularies
TOFHLA: Test of Functional Health Literacy in Adults
