# Supplementary material for: Effects of Individual Health Topic Familiarity on Activity Patterns During Health Information Searches
Source: JMIR Med Inform. 2015 Mar 17;3(1):e16. doi: 10.2196/medinform.3803 (PMC4381811; doi:10.2196/medinform.3803)
Supplement: Supplementary file 1 [file medinform_v3i1e16_app1.pdf]

Multimedia Appendix 1 Health Terminology Familiarity Survey

Section 1

Instruction

For each item below, check one option that is **most closely related** to the *italic* word. If you never heard the *italic* word/phrase before, please do not guess the answer and instead **select the unknown** option.

1. *Eczema*
   - Skin Inflammation
   - Broken bone
   - Stomach problem
   - Movement Disorder
   - Unknown

2. *Topical ointment*
   - Protein
   - Hormone
   - Surgery
   - Medicine
   - Unknown

3. *Heart attack*
   - Heart and blood vessel disease
   - Digestive system disease
   - Kidney disease
   - Endocrine disease
   - Unknown

4. *Automated External Defibrillator*
   - First aid medical device
   - Medical measurement device
   - Surgical instrument
   - Body scanner device
   - Unknown

5. *Urinalysis*
   - Disease
   - Medical test
   - Hormone
   - Enzyme
   - Unknown

6. *Urine specific gravity*
   - pH test
   - Concentration test
   - Protein test
   - Glucose test
   - Unknown

7. *Cholesterol*
   - Food allergy
   - Fat substance

Surgical procedure

Disease

Detoxification

Protein

Vaccine

Oral medication

Unknown

Unknown

8. Simvastatin

Section 2

Instruction

Select the correct answer for each question below.
If you never heard the *italic* word/phrase before, please do not guess the answer and instead **select the unknown** option.

1. If you are diagnosed with *eczema*, it means that …

   your skin shows patches of itchy, redness, and thickened area
   you have a broken bone in your wrist
   the inside lining of your stomach is wounded
   your tendon or muscle in the knee joint is injured
   Unknown

2. A *topical ointment* is …

   a type of protein for building muscle tissue and repairing damaged tissues
   a body chemical that responses to allergy or infection
   a diagnostic test involving the removal of sample tissue
   a type medication that is applied to the skin to reduce the inflammation
   Unknown

3. You frequently find brochures in the clinic or hospital about how to recognize a *heart attack*. To you heart attack means …

   the heart suddenly stops beating unexpectedly
   the artery that carries blood to the heart is blocked
   heartbeat rhythm problem, the heart may beat too fast, too slow, or too irregularly
   a damage to the heart muscle
   Unknown

4. The medical kit box (AED) as in the picture below is located in many public places. This kit is used to …

deliver electric shocks to a patient’s heart in

a sudden heartbeat stop incident

measure blood sugar level

filter harmful waste, salt, and excess fluid from the blood

determine the severity of injuries by scanning the affected body parts

Unknown

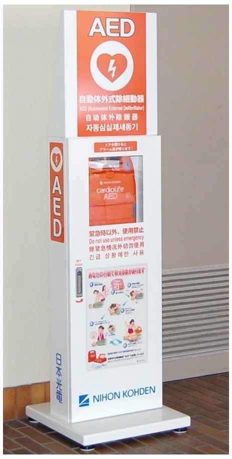

5. *Urinalysis* refers to …

disorder in the kidney and urine tract system

medical test that examines the physical, chemical, and microscopic properties of urine

hormone system that regulates the balance of blood pressure and water

enzyme that breaks down protein into smaller particle, e.g. amino acid

Unknown

6. *Urine density test* measures …

the level of acid in a urine

the concentration of substances in a urine

the excess amount of protein found in a urine sample

the amount of sugar found in a urine sample

Unknown

7. Too much *bad cholesterol* in the blood is dangerous because …

it may damage liver

it leads to kidney stone formation within the kidney or in the urinary tract

it can obstruct the absorption of good nutrients in the small intestine

it leads to artery blockage and increases heart attack risk

Unknown

8. *Simvastatin* is mainly prescribed for …

reducing total cholesterol level

the treatment of mild to moderate pain, inflammation and fever

lowering blood pressure level

the treatment of nasal congestion and runny nose from allergy

Unknown

Section 3

Instruction

Select the correct answer for each question below.
If you never heard the *italic* word/phrase before, please do not guess the answer and instead **select the unknown** option.

1. If you are diagnosed with *atopic dermatitis*, it means that …

   the inside lining of your stomach is wounded
   your skin shows patches of itchy, redness, and thickened area
   your tendon or tissue in the knee joint is injured
   you have fracture(s) in your wrist
   Unknown

2. A *topical corticosteroid* is …

   a type of protein for building muscle tissue and repairing damaged tissues
   a body chemical that responses to allergy or infection
   a diagnostic test that involves taking a sample of tissue for an examination under a microscope
   a type of drug to reduce inflammation and thickening of the skin
   Unknown

3. You frequently find brochures in the clinic or hospital about how to recognize a *myocardial infarction (MI)*. MI means …

   a heart condition in which the heart suddenly and unexpectedly stops beating
   a blockage in the artery that carries blood to the heart
   heartbeat rhythm problem, the heart may beat too fast, too slow, or too irregularly
   a damage to the heart muscle
   Unknown

4. An Automated External Defibrillator is a portable device to …

   deliver electric shocks to a patient's heart in a sudden cardiac arrest incident
   measure the approximate concentration of glucose in the blood
   filter harmful waste, salt, and excess fluid from the blood
   determine the severity of injuries by scanning the affected body parts
   Unknown

5. *Routine and Microscopy (R&M)* refers to …

disorder in the kidney and urine tract system

medical test that examines the physical, chemical, and microscopic properties of urine

hormone system that regulates the balance of blood pressure and water

enzyme that breaks down protein into smaller particle, e.g. amino acid

Unknown

6. *Urine specific gravity measures* …

how acidic or alkaline the urine is

the concentration of all chemical particles in the urine

the excess amount of protein found in a urine sample

the amount of glucose found in a urine sample

Unknown

7. High level of *low-density lipoprotein* may cause …

damage to the liver

the formation of kidney stone within the kidney or in the urinary tract

disorder in small intestine function to absorb good nutrients from food

artery blockage that can increase coronary disease risk

Unknown

8. *Statins* or *HMG COa Reductase Inhibitors* are drugs used to …

reduce blood cholesterol level

treat mild to moderate pain, inflammation and fever

lower blood pressure level

relieve nasal congestion and reduce the symptoms of an allergic reaction

Unknown
