# Supplementary material for: Effects of Individual Health Topic Familiarity on Activity Patterns During Health Information Searches
Source: JMIR Med Inform. 2015 Mar 17;3(1):e16. doi: 10.2196/medinform.3803 (PMC4381811; doi:10.2196/medinform.3803)
Supplement: Supplementary file 2 [file medinform_v3i1e16_app2.pdf]

Multimedia Appendix 2 Frequency and Proportion of Search Activity Type

Search	Frequency (*N*)			Proportion (%)		
Activity Type	L1	L2	L3	L1	L2	L3
Q-AccSE	196	115	83	8.09	9.55	8.6
Q-AccHW	4	2	12	0.17	0.17	1.2
Q-NewQ	78	65	57	3.22	5.40	5.9
Q-ModQ	162	75	48	6.68	6.23	5.0
E-ExamSR	298	170	131	12.29	14.12	13.6
E-DisSR	34	19	10	1.40	1.58	1.0
E-EvalI	565	260	208	23.31	21.59	21.5
E-FindQ	21	15	9	0.87	1.25	0.9
A-SelHI	295	159	158	12.17	13.21	16.3
A-SelGI	107	52	27	4.41	4.32	2.8
A-AccF	129	34	28	5.32	2.82	2.9
A-AccB	72	28	10	2.97	2.33	1.0
U-UseHI	139	107	96	5.73	8.89	9.9
U-UseGI	38	17	8	1.57	1.41	0.8
D-DisHI	187	47	57	7.71	3.90	5.9
D-DisGI	59	26	17	2.43	2.16	1.8
D-UnchkHI	28	7	5	1.16	0.58	0.5
D-UnchkGI	12	6	3	0.50	0.50	0.3
Total	2424	1204	967	100.00	100.00	100.0
