# Supplementary material for: Effects of Individual Health Topic Familiarity on Activity Patterns During Health Information Searches
Source: JMIR Med Inform. 2015 Mar 17;3(1):e16. doi: 10.2196/medinform.3803 (PMC4381811; doi:10.2196/medinform.3803)
Supplement: Supplementary file 3 [file medinform_v3i1e16_app3.pdf]

Multimedia Appendix 3 Most Frequent 5-gram Sequence Patterns in Each Familiarity Group

Top 20 frequent 5-gram sequence patterns in group L1

Rank.	Pattern	Occurrence Frequency	Percentage (%)[a]
1	A:AccSE A:ModQ E:ExamSR A:SelHI E:EvalI	55	5.8
2	E:EvalI D:DisHI A:AccSE A:ModQ E:ExamSR	35	3.7
3	A:ModQ E:ExamSR A:SelHI E:EvalI U:UseHI	27	2.9
4	E:EvalI D:DisHI E:ExamSR A:SelHI E:EvalI	26	2.8
5	A:ModQ E:ExamSR A:SelHI E:EvalI D:DisHI	24	2.6
6	D:DisHI A:AccSE A:ModQ E:ExamSR A:SelHI	23	2.4
**7**	E:EvalI U:UseHI A:AccSE A:ModQ E:ExamSR	23	2.4
8	E:EvalI A:XplorF E:EvalI A:XplorF E:EvalI	22	2.3
9	E:EvalI D:DisHI E:EvalI D:DisHI E:EvalI	22	2.3
10	E:ExamSR A:SelHI E:EvalI D:DisHI A:AccSE	22	2.3
11	E:ExamSR A:SelHI E:EvalI D:DisHI E:ExamSR	21	2.2
12	A:SelHI E:EvalI D:DisHI A:AccSE A:ModQ	20	2.1
13	A:SelHI E:EvalI D:DisHI E:ExamSR A:SelHI	18	1.9
14	A:AccSE A:NewQ E:ExamSR A:SelHI A:SelHI	17	1.8
15	E:ExamSR A:SelHI E:EvalI U:UseHI	17	1.8

Ran k.	Pattern	Occurrence Frequency	Percentage (%)[a]
	A:AccSE		
16	A:SelHI E:EvalI U:UseHI A:AccSE A:ModQ	17	1.8
17	D:DisHI E:ExamSR A:SelHI E:EvalI D:DisHI	16	1.7
18	U:UseHI A:AccSE A:ModQ E:ExamSR A:SelHI	16	1.7
19	A:AccSE A:NewQ E:ExamSR A:SelHI E:EvalI	15	1.6
20	A:ModQ E:ExamSR E:DisSR A:ModQ E:ExamSR	15	1.6
	Total Top 20 frequent patterns	451	48.0

[a] Total number of all 5-gram sequences = 940

Top 20 frequent 5-gram sequence patterns in group L2

Rank.	Pattern	Occurrence Frequency	Percentage (%)[a]
1	A:AccSE A:ModQ E:ExamSR A:SelHI E:EvalI	24	5.4
2	E:EvalI U:UseHI A:AccSE A:ModQ E:ExamSR	24	5.4
3	A:ModQ E:ExamSR A:SelHI E:EvalI U:UseHI	23	5.2
4	A:AccSE A:NewQ E:ExamSR A:SelHI E:EvalI	21	4.7
5	U:UseHI A:AccSE A:ModQ E:ExamSR A:SelHI	21	4.7
6	E:ExamSR A:SelHI E:EvalI U:UseHI A:AccSE	20	4.5
**7**	A:SelHI E:EvalI U:UseHI A:AccSE A:ModQ	17	3.8
8	A:NewQ E:ExamSR A:SelHI E:EvalI U:UseHI	16	3.6
9	A:AccSE A:NewQ E:ExamSR A:SelHI A:SelHI	13	2.9
10	A:AccSE A:ModQ E:ExamSR A:SelHI A:SelHI	10	2.3
11	A:AccSE A:NewQ E:ExamSR A:SelGI E:EvalI	10	2.3
12	E:ExamSR E:DisSR A:ModQ E:ExamSR A:SelHI	10	2.3
13	E:ExamSR A:SelHI A:SelHI E:EvalI E:EvalI	10	2.3
14	A:ModQ E:ExamSR E:DisSR A:ModQ E:ExamSR	9	2.0
15	E:ExamSR A:SelHI A:SelHI A:SelHI E:EvalI	8	1.8
16	A:ModQ E:ExamSR A:SelGI E:EvalI D:DisGI	8	1.8
17	A:ModQ E:ExamSR A:SelHI E:EvalI D:DisHI	8	1.8
18	A:NewQ E:ExamSR A:SelHI A:SelHI	8	1.8

Rank.	Pattern	Occurrence Frequency	Percentage (%)[a]
	E:EvalI		
19	A:AccSE A:ModQ E:ExamSR E:DisSR A:ModQ	7	1.6
20	A:AccSE A:ModQ E:ExamSR A:SelGI E:EvalI	7	1.6
	Total Top 20 frequent patterns	274	61.8

[a] Total number of all 5-gram sequences = 444

Top 20 frequent 5-gram sequence patterns in group L3

Rank.	Pattern	Occurrence Frequency	Percentage (%)[a]
1	A:AccSE A:NewQ E:ExamSR A:SelHI E:EvalI	18	5.0
2	A:AccSE A:ModQ E:ExamSR A:SelHI E:EvalI	17	4.7
3	A:NewQ E:ExamSR A:SelHI E:EvalI U:UseHI	16	4.5
4	E:EvalI D:DisHI A:AccSE A:ModQ E:ExamSR	12	3.3
5	A:ModQ E:ExamSR A:SelHI E:EvalI U:UseHI	11	3.1
6	A:AccSE A:NewQ E:ExamSR A:SelHI A:SelHI	10	2.8
**7**	E:ExamSR A:SelHI E:EvalI U:UseHI E:ExamSR	10	2.8
8	E:ExamSR A:SelHI A:SelHI A:SelHI E:EvalI	10	2.8
9	U:UseHI A:AccSE A:ModQ E:ExamSR A:SelHI	10	2.8
10	D:DisHI A:AccSE A:ModQ E:ExamSR A:SelHI	9	2.5
11	E:EvalI U:UseHI A:AccSE A:ModQ E:ExamSR	9	2.5
12	E:ExamSR A:SelHI E:EvalI D:DisHI A:AccSE	9	2.5
13	A:SelHI E:EvalI D:DisHI A:AccSE A:ModQ	9	2.5
14	A:SelHI E:EvalI U:UseHI A:AccSE A:ModQ	9	2.5
15	A:SelHI E:EvalI U:UseHI E:ExamSR A:SelHI	9	2.5
16	A:AccHW A:NewQ E:ExamSR A:SelHI E:EvalI	8	2.2
17	E:ExamSR A:SelHI E:EvalI A:XplorF E:EvalI	8	2.2
18	E:ExamSR A:SelHI E:EvalI U:UseHI	8	2.2

Rank.	Pattern	Occurrence Frequency	Percentage (%)[a]
	A:AccSE		
19	A:AccSE A:ModQ E:ExamSR A:SelHI A:SelHI	7	2.0
20	A:SelHI A:SelHI E:EvalI D:DisHI E:EvalI	7	2.0
	Total Top 20 frequent patterns	206	57.4

[a] Total number of all 5-gram sequences = 359
